# Two-Stage Hybrid Model for Efficiency Prediction of Centrifugal Pump

**DOI:** 10.3390/s22114300

**Published:** 2022-06-06

**Authors:** Yi Liu, Zhaoshun Xia, Hongying Deng, Shuihua Zheng

**Affiliations:** Institute of Process Equipment and Control Engineering, Zhejiang University of Technology, Hangzhou 310023, China; yliuzju@zjut.edu.cn (Y.L.); 2111902151@zjut.edu.cn (Z.X.); dhyty@zjut.edu.cn (H.D.)

**Keywords:** centrifugal pump efficiency, hybrid model, affinity law, Gaussian process regression

## Abstract

Accurately predict the efficiency of centrifugal pumps at different rotational speeds is important but still intractable in practice. To enhance the prediction performance, this work proposes a hybrid modeling method by combining both the process data and knowledge of centrifugal pumps. First, according to the process knowledge of centrifugal pumps, the efficiency curve is divided into two stages. Then, the affinity law of pumps and a Gaussian process regression (GPR) model are explored and utilized to predict the efficiency at their suitable flow stages, respectively. Furthermore, a probability index is established through the prediction variance of a GPR model and Bayesian inference to select a suitable training set to improve the prediction accuracy. Experimental results show the superiority of the hybrid modeling method, compared with only using mechanism or data-driven models.

## 1. Introduction

Centrifugal pumps are widely used in construction, municipal water supply and drainage, petroleum and chemical industries, thermal power and other industries [1,2]. Most pumps are driven by motors and their electricity consumption is huge [3,4]. Due to the high energy consumption of centrifugal pumps, the frequency conversion technology has been widely adopted to adjust the speed of centrifugal pump by means of a frequency converter, thus plays an important role in energy saving in the pump industry [5].

When the change rate of rotation speed does not exceed about 33% of the rated centrifugal speed, the change in efficiency can be ignored [6]. This approximation only means that the centrifugal pump curve points will maintain the same efficiency, while the pump will not operate at the same efficiency once inserted into the system. In fact, the operating point of a centrifugal pump is defined by the intersection of the centrifugal pump curve and the system curve. Consequently, the overall efficiency depends not only on the efficiency of the centrifugal pump itself, but also by the influence of the system [7]. If the operating efficiency of centrifugal pumps at different speeds can be accurately predicted, the energy-saving effect of centrifugal pumps under variable frequency conditions will be noticeable. Meanwhile, the centrifugal pump can maintain a suitable operating condition and extend its effective service life.

Traditionally, predicting the state of centrifugal pumps at different speeds mainly uses the affinity law of pump and the computational fluid dynamics (CFD) software. However, the affinity law of pump assumes that the efficiency of a pump is approximately constant at different speeds. In fact, the volumetric efficiency, hydraulic efficiency, and mechanical efficiency will also change when the rotational speed of a centrifugal pump changes [7,8]. Researchers have attempted to correlate the speed of centrifugal pump with the efficiency to make the prediction more accurate [9,10,11,12]. However, some assumptions include approximate values, especially ignoring the friction loss of pipeline system [7,13,14,15,16]. Additionally, the flow rate change at the same speed is mainly adjusted by the outlet throttle valve of system, and the friction loss of pipeline system changes constantly [12]. Therefore, it is still difficult to accurately describe the operating efficiency of a centrifugal pump only using the mechanism model. The accuracy of CFD method is dependent on the expertise of engineers, as it is sensitive to the mesh quality, the turbulence model, and the numerical schemes. Consequently, it is necessary to conduct the validation and verification for each simulation case [17]. Additionally, numerical simulation requires huge computational resources and computing time, which may not satisfy the requirement during the macro-control of the electric power systems [1,18].

Recently, several neural networks and data-driven empirical models (also named as soft sensors/analyzers) have been developed alternatively in the fluid machinery field [1,2,4,19,20,21,22,23,24,25,26,27]. Compared with mechanism models, the process data can reflect the characteristics without a sufficient understanding of the mechanism. As we know, the modeling accuracy of data-driven empirical models depends on the amount and reliability of modeling data. However, it is tricky to collect enough samples of different multiphase conditions due to the time-consuming and costly experiment process [28,29,30,31]. Thus, develop efficient strategies to enhance the prediction performance with limited data is necessary in data-driven empirical modeling (soft sensing) methods for pump characteristics.

Since both of mechanism and data-driven empirical models have pros and cons, the development of a hybrid model integrating both of their advantages is attractive in practice. Using the mechanism model, the prediction error is often acceptable because of the small friction loss of piping system in the large flow area of centrifugal pump operation [9]. However, the prediction is not accurate because of the large friction loss of piping system and centrifugal pump in the small flow area [8]. If the efficiency curve of different rotating speeds can be divided into two stages, i.e., the large flow one and the small flow one, only a data-driven model needs to be constructed for the small flow region. In such a situation, the requirement of large amount modeling data can be reduced, indicating that the hybrid model can be developed using limited samples.

When the valve opening is larger, the outlet flow of centrifugal pump is larger, and the friction loss of pipeline system is smaller, and vice versa [32,33]. Therefore, the valve opening can be utilized to divide the flow stages to the large flow one and small flow one. As a probabilistic data-driven modeling method, the Gaussian process regression (GPR) has been applied to evaluate the prediction uncertainty of a soft sensor model [34,35,36,37,38,39,40,41,42]. Using this feature of GPR model combined with Bayesian inference to form posterior probability, the difference in operating conditions of centrifugal pumps at different speeds can be measured by posterior probability, and a training set more similar with the test set can be found, thereby improving the predictive performance of a data-driven model.

In summary, the proposed hybrid model for the efficiency prediction of centrifugal pumps is so constructed. First, multiple GPR models are built according to different speeds to obtain the prediction variance, and the similarity between sample subsets at different rotational speeds is calculated. Then, according to the impact of valve opening on system friction loss and mechanism model prediction performance, the efficiency curve is processed in several sections. Finally, the efficiency of large flow stage is predicted using a mechanism model with the affinity law of pump, and a local GPR (LGPR) model is constructed to predict the efficiency of small flow stage.

The remainder of this work is so structured. In Section 2, efficiency curves and probabilistic modeling analysis at several typical speeds are discussed. In Section 3, the hybrid model is proposed to predict the efficiency of centrifugal pumps at variable speeds. In Section 4, prediction results of the hybrid model are compared with the experimental data to verify its effect. In Section 5, the work is summarized.

## 2. Experimental System and Process Analysis

### 2.1. Experimental System

**Figure 1:**
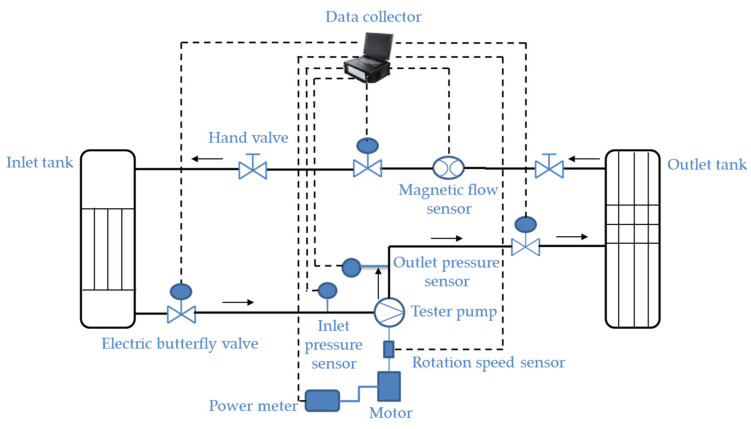
The diagram of experimental system for measuring the centrifugal pump efficiency.

**Figure 2:**
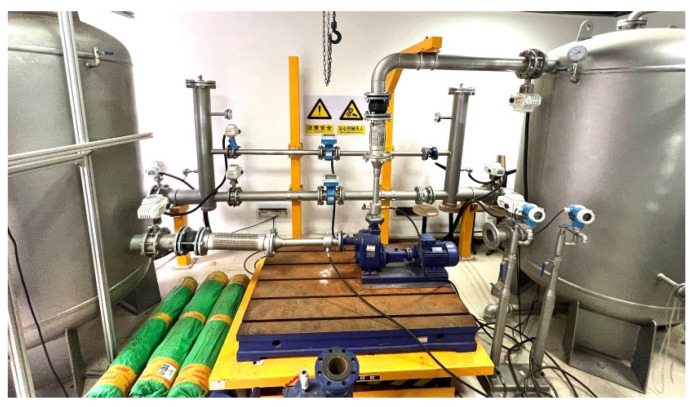
The experimental system for measuring the centrifugal pump efficiency.

The diagram of this experimental system is shown in Figure 1. In order to obtain the efficiency curves at different speeds, the ZW1150-20-20 self-priming centrifugal pump shown in Figure 2 is used in the experiment. The instruments of this experimental system are listed in Table 1.

The centrifugal pump is driven by a variable frequency motor, and water flows into the system through the centrifugal pump. The operating speed of the centrifugal pump is changed through a variable frequency drive. At the same speed, adjust the outlet flow of the centrifugal pump through the opening of the outlet valve in the pipeline system. Under different valve openings *V*, according to the flowmeter, the pressure sensor, and rotation speed sensor record the outlet flow *Q*, the inlet pressure $P_{s}$, the outlet pressure $P_{d}$, and the rotational speed *n*, respectively. Additionally, the shaft power *N* can be obtained according to the power meter. The efficiency of different flow points is calculated according to Equation (1) [12].

(1) $$\eta =\frac{\rho gQH}{N},$$

where ρ is the density of transfer liquid; $H=\frac{P_{d}-P_{s}}{\rho g}+H_{st}$ is the head of the centrifugal pump, and *H_st_* is the static head of the system of the centrifugal pump.

**Figure 3:**
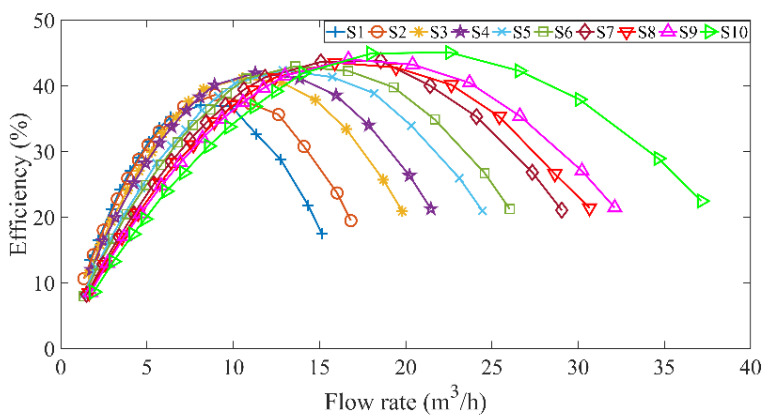
The centrifugal pump efficiency changes with the flow rate at different speeds.

Collect the experimental data by adjusting the frequency of variable frequency drive and the opening of outlet valve, then obtain the efficiency curves at different speeds according to Equation (1). A total of ten efficiency curves at different speeds (i.e., 1200 r/min, 1320 r/min, 1560 r/min, 1680 r/min, 1920 r/min, 2040 r/min, 2280 r/min, 2400 r/min, 2640 r/min, 2900 r/min for the datasets of $S_{1},S_{2},S_{3},S_{4},S_{5},S_{6},S_{7},S_{8},S_{9},S_{10}$) are collected from the experimental system including the efficiency curves at rated speeds, as shown in Figure 3.

### 2.2. Process Mechanism Analysis

The efficiency curves at different speeds have a common feature. As flow increases, the efficiency first increases rapidly and then decreases gradually, as shown in Figure 3. The main reason is that when the outlet valve opening is small, flow into the system is small, and the system loss is large. Deviating from the design flow of centrifugal pump results in a large impact loss inside the centrifugal pump. When the valve opening gradually increases, the efficiency of centrifugal pump will also increase. When the best efficiency point of centrifugal pump is reached, the efficiency of centrifugal pump gradually decreases as the flow rate increases. This is because the excessive flow also causes the centrifugal pump to deviate from the design flow, resulting in excessive shock loss inside the centrifugal pump, which in turn leads to a decrease in the efficiency of centrifugal pump [8,12].

Based on the common points of efficiency curves at different speeds, using the affinity law of pump, the problem of the change of efficiency with rotational speed is transformed into an empirical formula for the ratio of efficiency to rotational speed [6], as shown in Equation (2).

(2) $$\frac{1-\eta_{x}}{1-\eta_{e}}={\left(\frac{n_{e}}{n_{x}}\right)}^{m},$$

where $\eta_{e}$ represents the efficiency under the speed $n_{e}$ (rated speed of the centrifugal pump) and $\eta_{x}$ represents the efficiency under the speed of $n_{x}$ (required efficiency), and *m* = 0.1 is an empirical coefficient which can be obtained by the relationship between the efficiency ratio and the speed ratio [6].

However, Equation (2) contains approximate values, especially ignoring the friction loss of pipeline system. The system friction loss in the large flow region is small, so the efficiency prediction is relatively accurate [7,9]. However, the efficiency prediction is not accurate for the small flow region. Under different speeds, the efficiency curves have common characteristics mainly because of the affinity law of pump [11]. Generally, the similarity of pump operating conditions is described through the operating speed of pump. The closer the operating speed is to rated speed, the higher the similarity [11]. However, there is not a criterion to clearly measure the similarity of pump operation at different speeds. To this end, using the probability information of the GPR model, a criterion is established to measure the similarity between various operating conditions at different speeds, thus providing a reasonable training set for GPR.

### 2.3. Process Mechanism Analysis

One appealing property of the GPR model is that it can provide a confidence level with its variance. Generally, the GPR model approximates a training set $S=\left\{X,y\right\}={\left\{x_{i},y_{i}\right\}}_{i=1}^{N}$ with *N* training samples. The valve opening *V*, the outlet flow *Q*, the inlet pressure $P_{s}$, and the outlet pressure $P_{d}$ are selected as the input variables, i.e., $x_{i}={\left\{P_{si},P_{di},V_{i},Q_{i}\right\}}^{T}$. The actual efficiency is the output variable, i.e., $y_{i}=\eta_{i}$. For an output variable **y**, the GPR model is the regression function with a Gaussian prior distribution and zero mean or in a discrete form [34].

(3) $$y={\left(y_{i},\cdots ,y_{N}\right)}^{T}∼G\left(0,C\right),$$

where C is the N×N covariance matrix with the *ij*th element $C\left(x_{i},x_{j}\right)$. Using the Bayesian method to train the GPR model, the matrix C can be estimated. For a test sample set with $N_{t}$ input samples $X_{t}={\left\{x_{t,i}\right\}}_{i=1}^{N_{t}},t=1,\cdots ,T$, the output variable ${\hat{y}}_{t,i}$ and its variance $\sigma_{{\hat{y}}_{t,i}}^{2}$ can be calculated as follows [34]:

(4) $${\hat{y}}_{t,i}=k_{t,i}^{T}C^{-1}y,$$

(5) $$\sigma_{{\hat{y}}_{t,i}}^{2}=k_{t,i}-k_{t,i}^{T}C^{-1}k_{t,i},$$

where $k_{t,i}={\left[C\left(x_{t,i},x_{1}\right),C\left(x_{t,i},x_{2}\right),\cdots ,C\left(x_{t,i},x_{N}\right)\right]}^{T}$ is the covariance vector between the new input and the training data, and $k_{t,i}=C\left(x_{t,i},x_{t,i}\right)$ is the covariance of the new input [34].

Train multiple GPR soft sensor models separately through sample subsets at different speeds, and evaluate the relationship between a single GPR model and the test sample set $X_{t}={\left\{x_{t,i}\right\}}_{i=1}^{N_{t}},t=1,\cdots ,T$. The mean of the posterior probability $P\left({\mathrm{GPR}}_{l}|x_{t}\right)$ , which is comprised by the prediction variance $\sigma_{{\hat{y}}_{t,i}}^{2}$ and Bayesian theorem in the GPR model, is used to measure similarity of datasets at different speeds. The index is defined as:

(6) $$P\left({\mathrm{GPR}}_{l}|x_{t}\right)=\overset{N_{t}}{\underset{i=1}{\sum }}\frac{P\left(x_{t,i}|{\mathrm{GPR}}_{l}\right)}{P\left(x_{t,i}\right)}=\overset{N_{t}}{\underset{i=1}{\sum }}\frac{N_{t}}{v_{l,x_{ti}}\overset{L}{\underset{l=1}{\sum }}\left(N_{l}/v_{l,x_{ti}}\right)},l=1,\cdots ,L,$$

The mean ensemble posterior probability (MEPP) be defined as:

(7) $$MEPP_{l,t}=\frac{P\left({\mathrm{GPR}}_{l}|X_{t}\right)}{N_{t}}=\overset{N_{t}}{\underset{i=1}{\sum }}\frac{N_{l}}{v_{l,x_{t,i}}N_{t}\overset{L}{\underset{l=1}{\sum }}\left(N_{l}/v_{l,x_{t,t}}\right)}100,l=1,\cdots ,L,$$

where $N_{l}$ represents the number of samples in the training sample subset; $N_{t}$ represents the number of samples in the test set; $v_{l,x_{t,i}}=\frac{\sigma_{{\hat{y}}_{t,i}}^{2}}{\left|{\hat{y}}_{l}\right|}\times 100\%,\;l=1,\cdots ,L$; $\sigma_{{\hat{y}}_{t,i}}^{2}$ represents ${\mathrm{GPR}}_{l}$ model’s prediction uncertainty for $x_{t,i}$.

A larger value of ${\mathrm{MEPP}}_{l,t}$ indicating a larger $P\left({\mathrm{GPR}}_{l}|x_{t}\right)$, so the test set $X_{t}$ is more suitable to be predicted by the ${\mathrm{GPR}}_{l}$ model. It means more similar between the training set $X_{l}$ for training the ${\mathrm{GPR}}_{l}$ model for the test set $X_{t}$. When a test set with a new speed appears, the MEPP index is used to find similar training sample subsets to form a training set for GPR to predict the efficiency of the test set. Additionally, in order to reduce the excessive dependence on the experimental data and reduce the experimental burden, the mechanism model based on the affinity law of pump is combined. Consequently, a hybrid modeling method is proposed to predict the efficiency of centrifugal pump at different flow stages.

## 3. Proposed Two-Stage Hybrid Model

A two-stage hybrid modeling method is used to construct an integrated soft sensor model to predict the efficiency of centrifugal pump at different speeds. By analyzing the impact of valve opening on efficiency, the efficiency curve is segmented. Sequentially, the efficiency of different flow stages can be predicted using data-driven model and mechanism model in an auto-switched manner.

### 3.1. Process Mechanism Analysis

The flow adjustment at the same speed mainly depends on the outlet throttle valve of the system [15]. The head curve of the system can be obtained from the knowledge of pipes and static head through simple hydraulic laws. The head curve of a general water supply system can be defined as [32]:

(8) $$H_{p}=H_{st}+KQ^{2},$$

where $H_{p}$ represents the piping system head, and *K* represents the dynamic head coefficient (friction loss).

**Figure 4:**
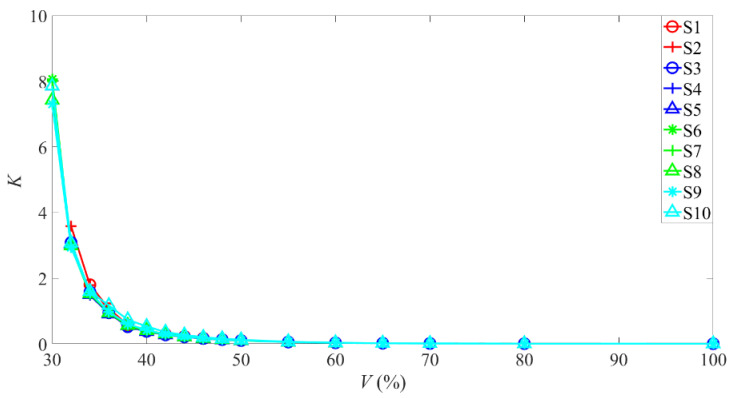
The curve of *K* values with valve opening at different speeds.

As shown in Figure 4, the curve of *K* values with valve opening at different speeds show two common characteristics. The first is, as the valve opening continues to increase, the *K* value decreases sharply, and when the valve opening is about 50%, the *K* value is close to zero. The second is, when the valve opening is between 30% and 50%, the *K* value of the same valve opening is not the same at different speeds. Therefore, for simplicity, the stage where the valve opening is larger than 50% is defined as a large flow stage, and the stage where the valve opening is less than 50% is defined as a small flow stage.

The training sample set $S=\left\{X,y\right\}={\left\{x_{i},y_{i}\right\}}_{i=1}^{N}$ and the test sample set $X_{t}={\left\{x_{t,i}\right\}}_{i=1}^{N_{t}}$ are divided into two stages by the size of the valve opening. For convenience, redefine the training sample set as $S=\left(S_{m},S_{h}\right)$ and the test sample set as $X_{t}=\left(X_{t,m},X_{t,h}\right)$. Among them, $S_{m}$ is the training sample set of the small flow stage, $S_{h}$ is the training sample set of the large flow stage, $X_{t,m}$ is the test sample set of the small flow stage, and $X_{t,h}$ is the test sample set of the large flow stage.

### 3.2. Stage Modeling Method

**Figure 5:**
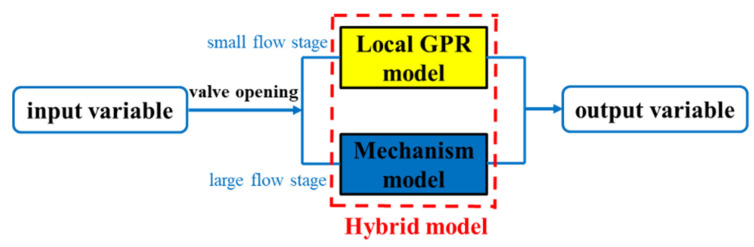
Hybrid model for prediction of the centrifugal pump efficiency.

The two-stage hybrid modeling method can be implemented as follows. In the small flow stage, due to the influence of the system friction loss, the prediction result of the mechanism model is not accurate. Additionally, the opening of the outlet valve is small, the pressure difference between the inside and outside of the valve is large, and the valve opening is more sensitive to the change of flow, so more samples can be obtained. Therefore, according to the MEPP index in Equation (7), the LGPR model is trained using suitable sample sets of the small flow stage and it is used to predict the efficiency in this stage. This is different from the GPR model that is constructed using samples from the whole flow stage. In the large flow stage, the friction loss of system is small, and the mechanism model is used to predict the efficiency. First judge whether the test sample belongs to the large flow stage or the small flow stage according to the valve opening. As shown in Figure 5, if it belongs to the small flow stage, use the LGPR model to predict according to Equation (4); if it belongs to the large flow stage, adopt Equation (2) to predict.

**Figure 6:**
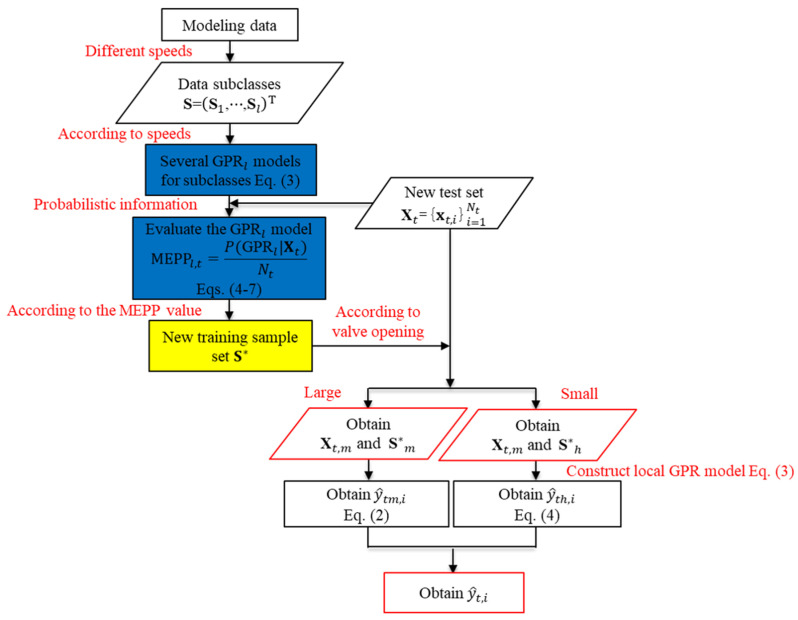
Modeling flowchart for the efficiency prediction of centrifugal pump at different rotational speeds.

The proposed modeling method uses available process knowledge and model information. In summary, the main implemented steps are illustrated in Figure 6. The step-by-step procedures are described as follows.

Step 1: Collect data at different speeds of the centrifugal pump $S=\left\{X,y\right\}={\left\{x_{i},y_{i}\right\}}_{i=1}^{N}$.

Step 2: Train multiple GPR models according to the sample subsets at different speeds using Equation (3).

Step 3: For a test sample set at a new speed, $X_{t}$ is calculated by multiple GPR models using Equations (4)–(7) to obtain ${\mathrm{MEPP}}_{l,t}$, and select several training sample subsets with relatively larger GPR models of ${\mathrm{MEPP}}_{l,t}$ to form a new training sample set $S^{*}$.

Step 4: The test sample set $X_{t}$ and the new training sample set $S^{*}$ are segmented according to the valve opening to obtain a new test sample set $X_{t,m}$ and $X_{t,h}$, and a new training sample set $S_{m}^{*}$ and $S_{h}^{*}$.

Step 5: For the test sample set $X_{t,h}$ in the large flow stage, calculate the prediction efficiency using Equation (2). For the test sample set $X_{t,m}$ in the small flow stage, first train the LGPR model according to the training sample set $S_{m}^{*}$ using Equation (3), and calculate the prediction efficiency using Equation (4).

Step 6: Finally, the prediction efficiency of the two stages is integrated to obtain the prediction efficiency of the test sample set $X_{t}$.

In the above modeling steps, useful process knowledge and GPR-based probability information are effectively integrated into the hybrid model to predict the efficiency of centrifugal pumps at different speeds. Separate modeling at each stage can better handle the process with different characteristics, reduce dependence on experimental data, and improve the prediction accuracy. From engineering point of view, this two-stage hybrid modeling method can be implemented straightforward.

## 4. Experimental Results and Discussion

Under the operation conditions described in Section 2.1, altogether 165 samples of ten operation speeds denoted as $S=\left(S_{1},\cdots ,S_{10}\right)$ are collected from the experimental system shown in Figure 1. Six sets $\left(S_{1},S_{3},S_{5},S_{6},S_{9},S_{10}\right)$ are used for training and the remaining four sets $\left(S_{2},S_{4},S_{7},S_{8}\right)$ are for test. To compare the prediction performance of different models, two common performance indices, i.e., the root mean square error (RMSE) and the maximum absolute relative error (MARE), are adopted as follows:

(9) $${\mathrm{RMSE}}_{i}=\sqrt{\sum_{i=1}^{N_{t}}{\left({\hat{y}}_{t,i}-y_{t,i}\right)}^{2}/N_{t}},i=1,2;t=1,\cdots ,N_{t}$$

(10) $${\mathrm{MARE}}_{i}=\max\left|{\hat{y}}_{t,i}-y_{t,i}\right|/y_{t,i}\times 100\%,i=1,2;t=1,\cdots ,N_{t},$$

where ${\hat{y}}_{t,i}$ represents the predicted value of $y_{t,i}$.

**Figure 7:**
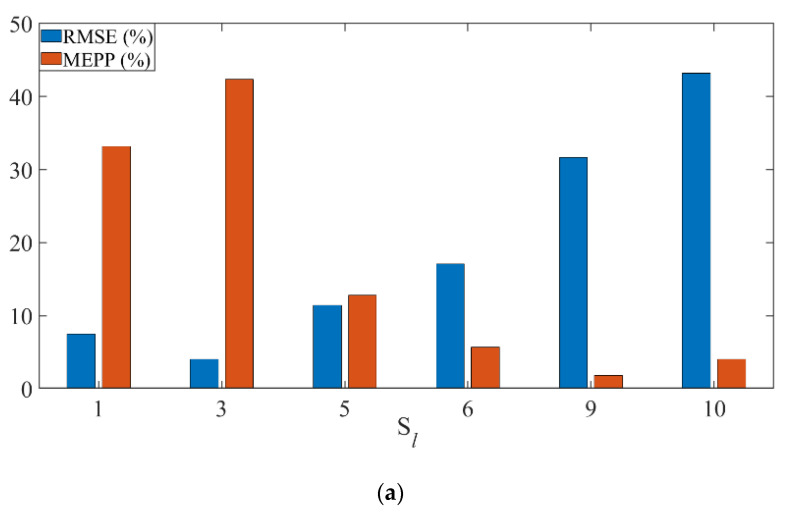

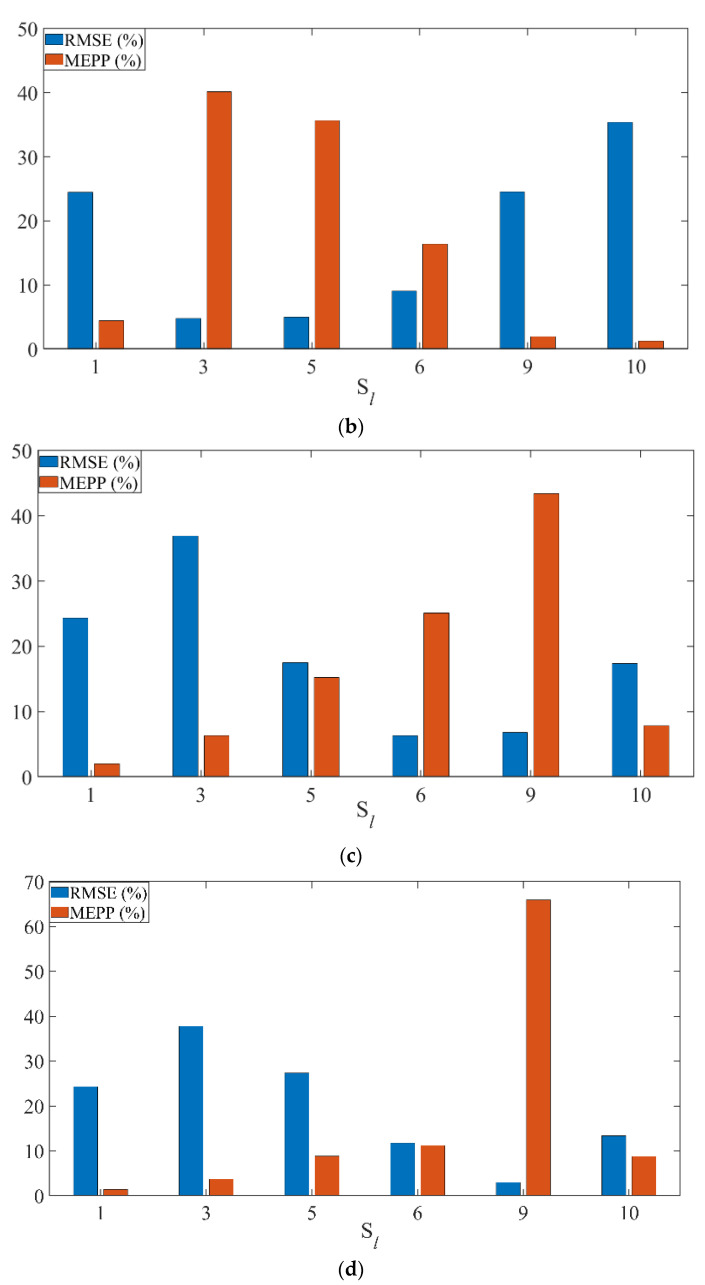
(**a**) GPR models trained by different sample subsets predict the test set $S_{2}$ to obtain the corresponding MEPP and RMSE values (**b**) GPR models trained by different sample subsets predict the test set $S_{4}$ to obtain the corresponding MEPP and RMSE values (**c**) GPR models trained by different sample subsets predict the test set $S_{7}$ to obtain the corresponding MEPP and RMSE values (**d**) GPR models trained by different sample subsets predict the test set $S_{8}$ to obtain the corresponding MEPP and RMSE values.

First, the effect of the MEPP index is verified. A larger value of MEPP means that the test sample set is more similar with the training sample subset of the training GPR model, thus the RMSE value is smaller. According to the four test sample sets of $S_{2}$, $S_{4}$, $S_{7}$ and $S_{8}$, the MEPP and RMSE values of the GPR models trained by the corresponding six sample subsets are shown in Figure 7. The results indicate that the similarity between the test sample set and the training sample subset can be measured by the MEPP index, and a new training set can be formed by selecting more similar subset $S^{*}$ to construct a suitable LGPR model at the small flow stage. For this case, the new training set of $S_{2}$ is $\left(S_{1},S_{3},S_{5}\right)$, the new training set of $S_{4}$ is $\left(S_{3},S_{5},S_{6}\right)$, the new training set of $S_{7}$ is $\left(S_{5},S_{6},S_{9}\right)$, and the new training set of $S_{8}$ is $\left(S_{6},S_{9},S_{10}\right)$, respectively.

**Figure 8:**
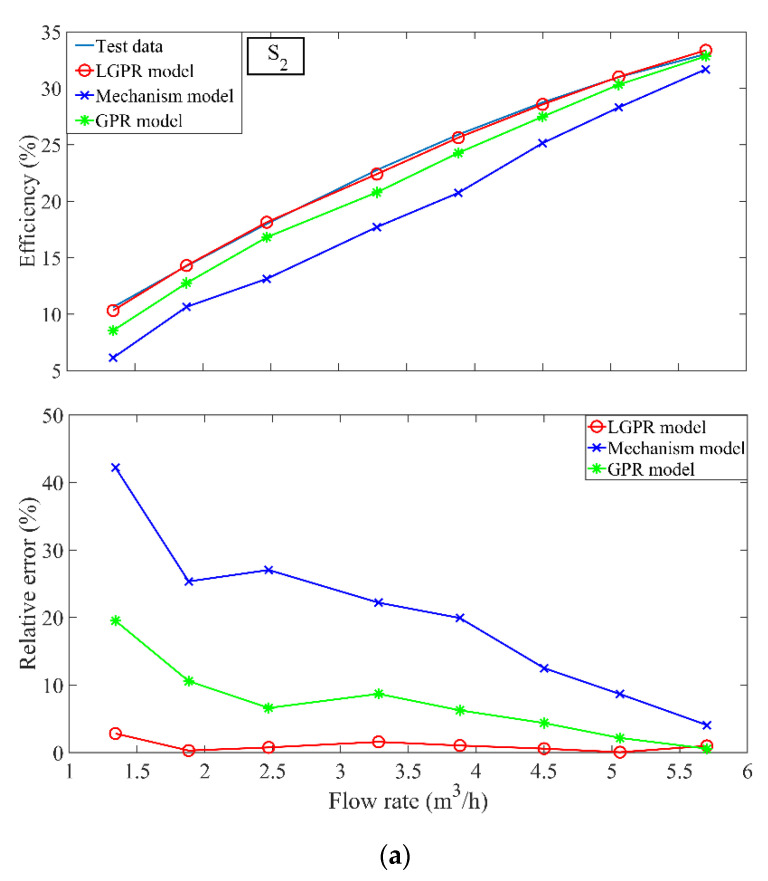

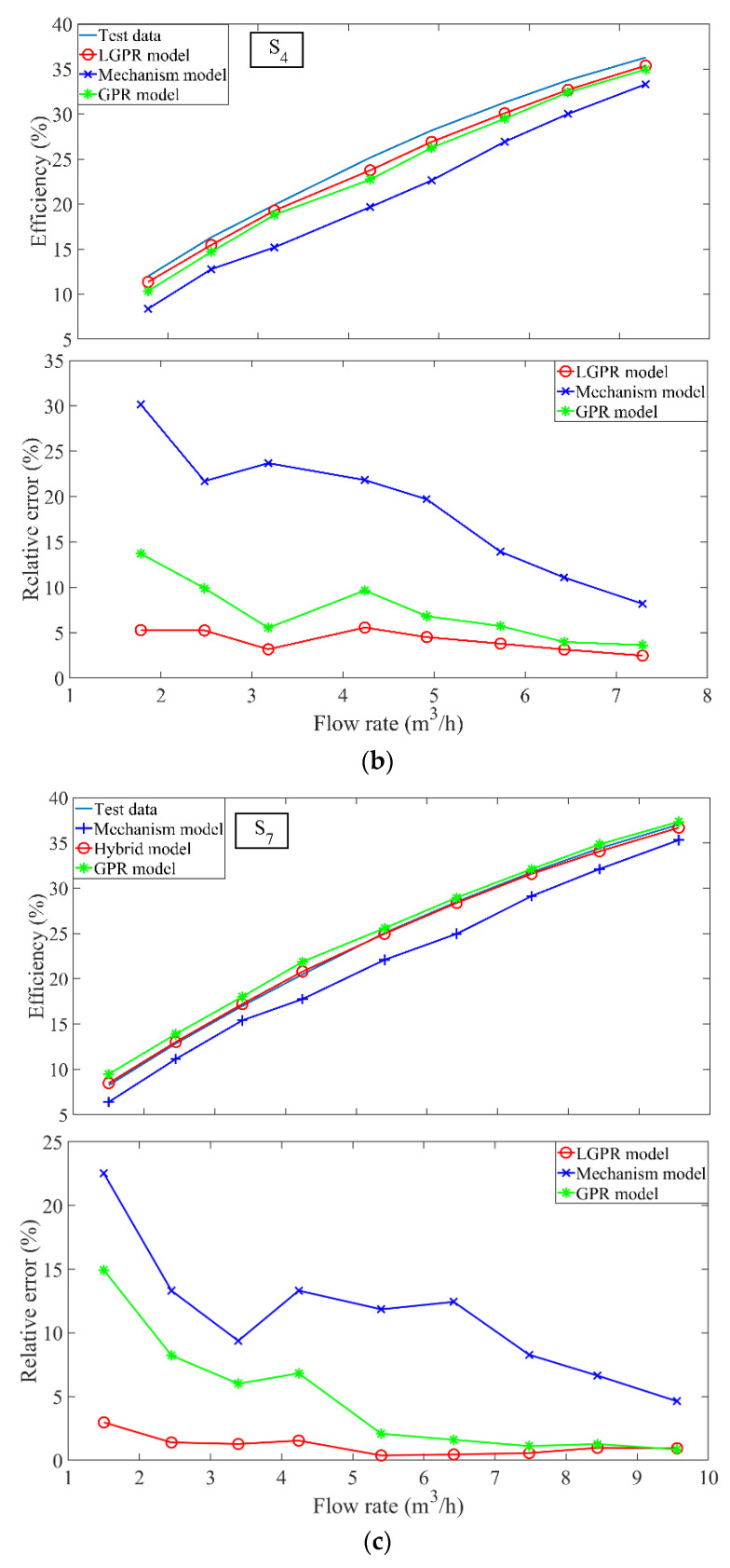

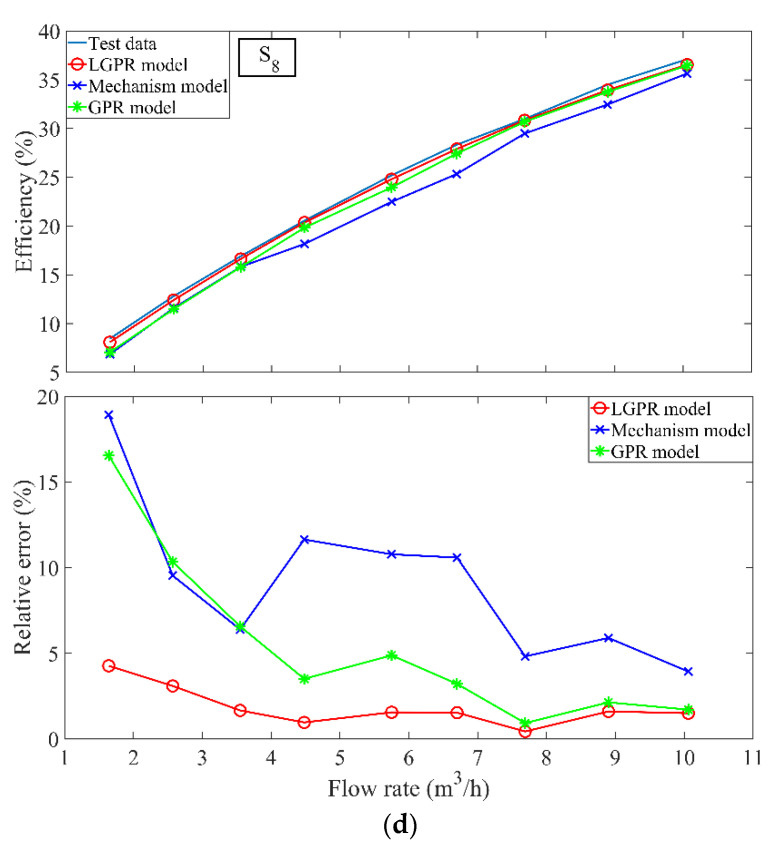
(**a**) The LGPR model and the GPR model prediction results and relative error of the small-flow stage of the test set $S_{2}$ (**b**) The LGPR model and the GPR model prediction results and relative error of the small-flow stage of the test set $S_{4}$ (**c**) The LGPR model and the GPR model prediction results and relative error of the small-flow stage of the test set $S_{7}$ (**d**) The LGPR model and the GPR model prediction results and relative error of the small-flow stage of the test set $S_{8}$.

Since the valve opening in the large flow stage is large, the friction loss of system is small. The efficiency of the large flow stage is predicted by the mechanism model based on the pump affinity law of pump. The prediction result of the small flow stage for the four test sample sets $S_{2}$, $S_{4}$, $S_{7}$, and $S_{8}$ are shown in Figure 8. Compared with the GPR model and the mechanism model, the training set $S^{*}$ is divided into two stages by the valve opening, and the training set $S_{m}^{*}$ in the small flow interval is used. Notice that the GPR and LGPR models are trained with different samples. The LGPR model has good prediction performance for the small flow stage. As also shown in Table 2, the MARE values of three models validate that the LGPR model can be used in the small flow stage.

**Figure 9:**
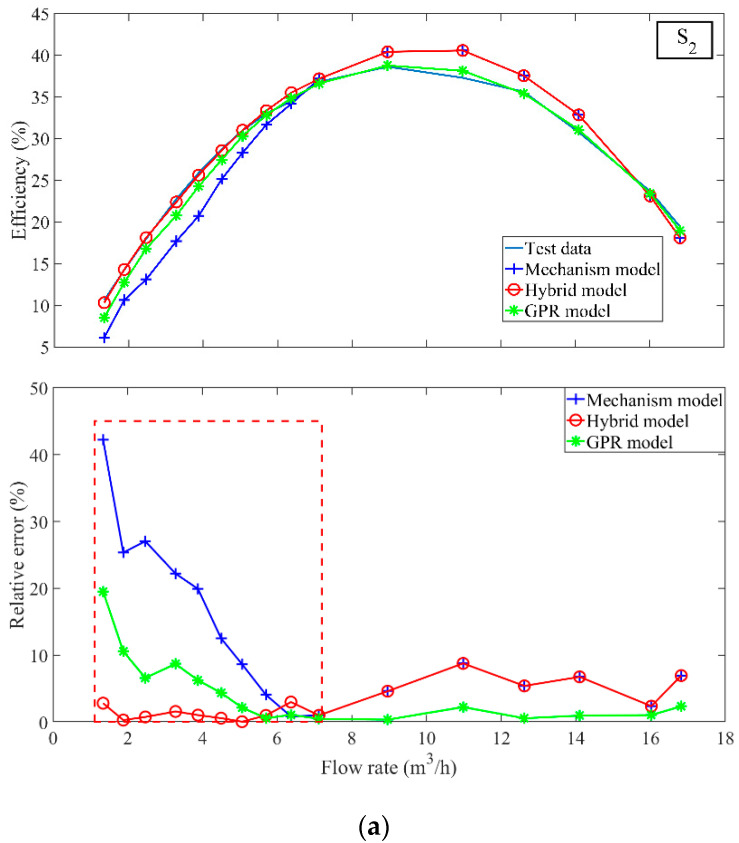

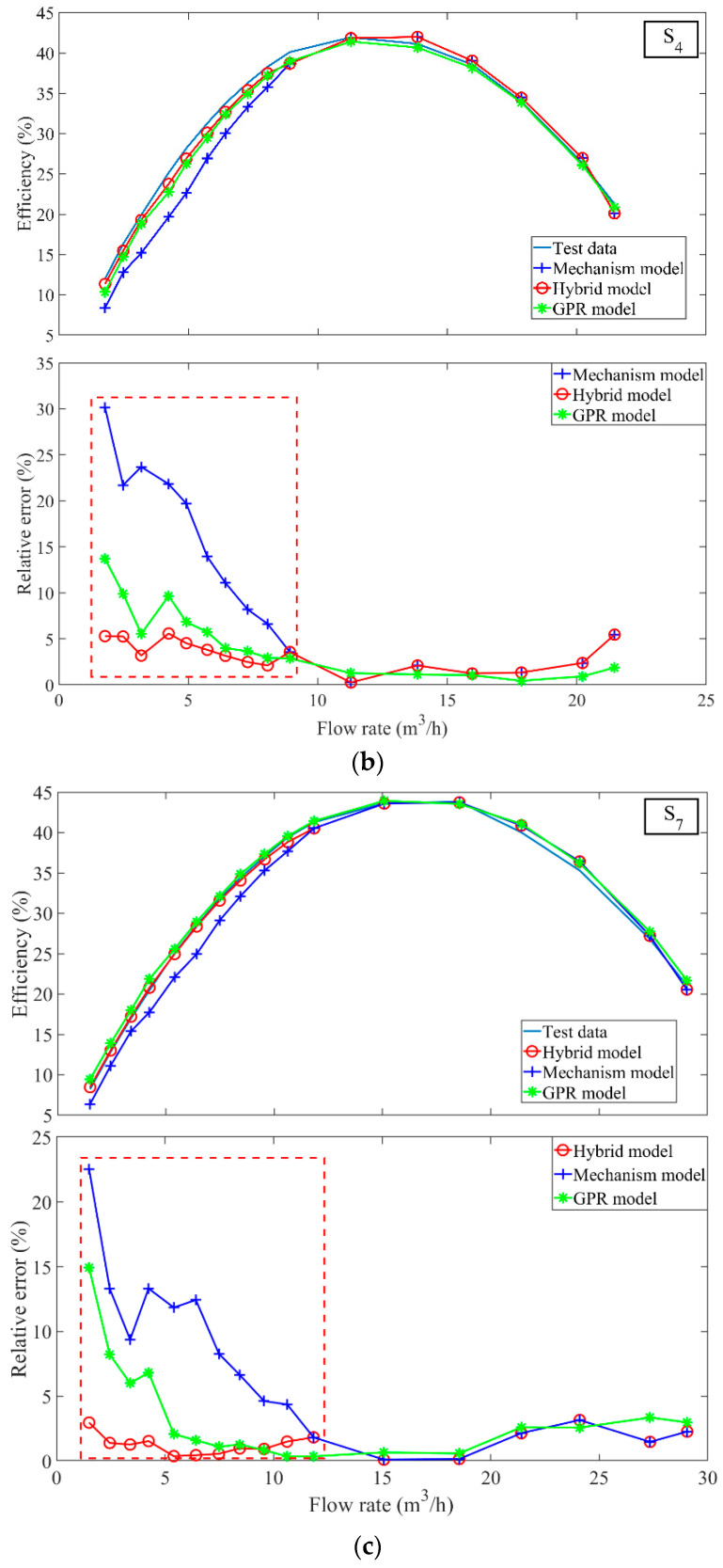

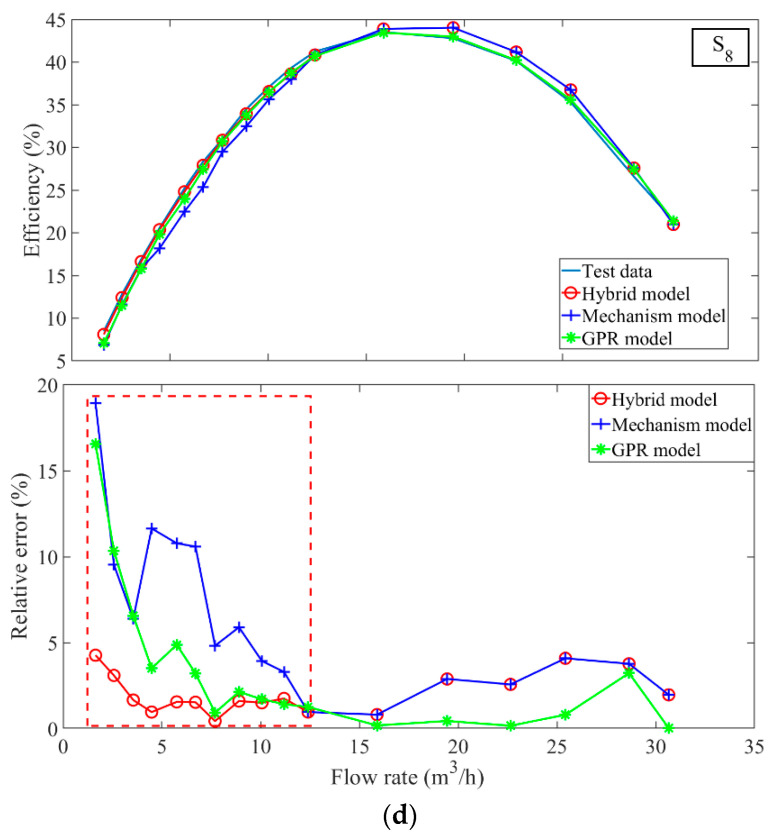
(**a**) The hybrid model, the GPR model and the mechanism model prediction results and relative error of the test set $S_{2}$ (**b**) The hybrid model, the GPR model and the mechanism model prediction results and relative error of the test set $S_{4}$ (**c**) The hybrid model, the GPR model and the mechanism model prediction results and relative error of the test set $S_{7}$ (**d**) The hybrid model, the GPR model and the mechanism model prediction results and relative error of the test set $S_{8}$.

The results of the two stages are integrated into the hybrid model to predict the efficiency of centrifugal pump at different speeds. For four different speeds, namely the test sample sets $S_{2}$, $S_{4}$, $S_{7}$, and $S_{8}$, the prediction results shown in Figure 9 indicate that the hybrid model can achieve a good prediction of the centrifugal pump efficiency.

The hybrid model, the GPR model, and the mechanism model based on the affinity law of pump are compared. Table 3 lists the performance comparison results of three models. Among them, the hybrid model has the best prediction effect, while the GPR and the mechanism models are inferior. As shown in Table 4, the mechanism model requires the least experimental data (for the efficiency prediction of $S_{2}$, $S_{4}$, $S_{7}$, and $S_{8}$ datasets using Equation (2)). The main reason is that the mechanism model requires only the efficiency points at rated speed with the same valve opening as the test datasets (i.e., $S_{2}$, $S_{4}$, $S_{7}$, and $S_{8}$). While the GPR model is purely data-driven and thus requires the most experimental data decided by the selected training samples (e.g., for $S_{2}$ the training set is $\left(S_{1},S_{3},S_{5}\right)$). The hybrid model combines LGPR for the small flow stage and the mechanism model for the large flow stage. Consequently, the required samples can be separately determined for each stage.

In summary, the hybrid model makes full use of the process knowledge of centrifugal pump, while avoiding the empirical error of the mechanism model, so it has better predictive performance. Compared with the GPR model, the hybrid model requires fewer samples, which reduces the experimental burden. Consequently, the hybrid model can be simply applied to practical centrifugal pumps.

## 5. Conclusions

This work proposes a hybrid knowledge-and-data soft sensor model to predict the efficiency of centrifugal pumps at different speeds. The GPR with its probabilistic inferencing method is utilized to select the suitable datasets to construct an appropriate data-driven prediction model for the low flow region. The advantages of mechanism and GPR models are combined, thus better prediction results can be obtained in different flow stages. Consequently, the hybrid model maintains the prediction accuracy and shows the simplicity because it reduces the number of modeling samples compared with a purely data-driven model. The experimental results validate its feasibility and simplicity. Some future research topics include how to improve the prediction accuracy in large flow stage and how to collect more informative experimental data in an active and efficient manner.

## Figures and Tables

**Table 1:** Information of measuring sensor.

Instrument	Measurement Range	Accuracy
Magnetic flow sensor	0~150 m^3^/h	0.2%
Outlet pressure sensor	0~4 MPa	0.05%
Inlet pressure sensor	−0.4~0.4 MPa	0.05%
Power meter	Current, Voltage and Power	0.5%
Rotation speed sensor	0~20,000 r/min	±1 r/min

**Table 2:** The MARE (%) values of the LGPR, GPR, and mechanism models of test sets.

	LGPR Model	GPR Model	Mechanism Model
$S_{2}$	2.79	20.02	43.26
$S_{4}$	5.27	14.65	29.91
$S_{7}$	3.12	15.06	23.82
$S_{8}$	4.72	17.12	18.62

**Table 3:** The RMSE values of the hybrid, GPR, and mechanism models of test sets $S_{2}$, $S_{4}$, $S_{7}$, $S_{8}$.

	Hybrid Model	GPR Model	Mechanism Model
$S_{2}$	0.88	1.06	3.21
$S_{4}$	0.76	1.28	3.28
$S_{7}$	0.48	0.76	1.86
$S_{8}$	0.60	0.78	1.60

**Table 4:** The number of samples required for test sets of the hybrid, GPR, and mechanism models.

	Hybrid Model	GPR Model	Mechanism Model
$S_{2}$	32	48	16
$S_{4}$	32	48	16
$S_{7}$	35	51	17
$S_{8}$	35	51	17

## Abbreviations

The following abbreviations are used in this paper:	
CFD	computational fluid dynamics
GPR	gaussian process regression
LGPR	local gaussian process regression
MARE	maximum absolute relative error
MEPP	mean ensemble posterior probability
RMSE	root-mean-square error
The following symbols are used in this paper:	
*H*	head, m
*H_p_*	piping system head, m
*H_st_*	static head, m
*K*	dynamic head coefficient
*N*	shaft power, kW
*n*	rated speed, r/min
*P_d_*	inlet pressure, MPa
*P_s_*	outlet pressure, MPa
*Q*	outlet flow, m^3^/h
*V*	valve opening,%
*ρ*	density, kg/m^3^
*η*	efficiency, %
